# Regularized Exploratory Bifactor Analysis With Small Sample Sizes

**DOI:** 10.3389/fpsyg.2020.00507

**Published:** 2020-04-09

**Authors:** Sunho Jung, Dong Gi Seo, Jungkyu Park

**Affiliations:** ^1^School of Management, Kyung Hee University, Seoul, South Korea; ^2^Department of Psychology, Hallym University, Chuncheon, South Korea; ^3^Department of Psychology, Kyungpook National University, Daegu, South Korea

**Keywords:** exploratory bifactor analysis, small sample size, unweighted least squares, regularized exploratory factor analysis, Monte Carlo simulation

## Abstract

Several methods of factor extraction have recently gained popularity as a procedure for dealing with estimation problems associated with small sample sizes, which can be found in the various behavioral science disciplines, such as comparative psychology and behavior genetics. Two popular approaches for particularly small samples (below 50) include unweighted least squares factor analysis (ULS-FA) and regularized exploratory factor analysis (REFA). However, it is unclear how well each of the approaches performs with small samples in the context of exploratory bifactor modeling. In the current study, a comprehensive simulation study was conducted to evaluate the small sample behavior of the two approaches in terms of bifactor structure recovery under different sample size, factor loading, number of variables per factor, number of factors, and factor correlation experimental conditions. The results show that REFA is recommended for use over ULS-FA, particularly in the conditions involving low factor loadings, few group factors, or a small number of variables per factor.

## Introduction

Researchers are often interested in revealing a multidimensional underlying structure of psychological construct. Bifactor model analysis is one of the major approaches for specifying several distinct but related dimensions that constitute the overall construct. The specification of a bifactor model represents factorial structures that include the general factor and one or more group factors; the first represents the broad common instrumental factor underlying all the items, and the latter includes domain-specific factors that are responsible for variance among particular sets of items. Previous studies have extensively demonstrated the benefits of using the bifactor model to analyze behavioral data (see Chen et al., 2006; Pomplun, 2007; Reise, 2012).

Recently, exploratory bifactor modeling has gained popularity for its ability to uncover bifactor structures, particularly in cases where researchers have insufficient *a priori* knowledge to specify the underlying factor patterns, or in the early phase of factor analytic research (Murray et al., 2016). Exploratory bifactor analysis using the Schmid-Leiman orthogonalization (Schmid and Leiman, 1957) is perhaps the most well-known procedure for elucidating a bifactor structure (e.g., Reise et al., 2010). This method makes it possible to partition item variance into two sources attributable to general and domain-specific factors and shed light on the systematic sources of item variance. The Schmid-Leiman bifactor exploratory factor analysis has been used in a variety of psychological researches, including in studies of intelligence (Dombrowski, 2014), personality (Chernyshenko et al., 2001), psychiatric disorders (Brouwer et al., 2013), and scale reduction (Ebesutani et al., 2012).

Despite the practical benefit of exploratory bifactor analysis in empirical applications, a small sample size issue remains unresolved, even studies with limited samples are common in social and behavioral research such as behavior genetics. Prior work has routinely recommended using unweighted least squares factor analysis (ULS-FA) as a viable method of factor extraction under small samples (e.g., Preacher and MacCallum, 2002; de Winter et al., 2009). Recent methodological advances have made it possible to incorporate the idea of regularization into factor analysis frameworks. Regularized exploratory factor analysis (REFA) (Jung and Takane, 2008; Jung and Lee, 2011) has been introduced as an alternative approach of conducting exploratory factor analysis with small sample sizes. The applications for REFA have been increasing rapidly in many fields such as behavior science due to its usefulness in handling the limited sample size problems, resulting in more stable parameter estimates (e.g., Wilson et al., 2018).

Although past studies have identified REFA as an effective alternative approach for overcoming the small sample size problem when exploring latent structures, the performance of REFA in conducting exploratory bifactor modeling has not yet been examined extensively. Therefore, the major purpose of this study was to extend REFA to exploratory bifactor modeling by incorporating the Schmid-Leiman procedure. We also set out to comprehensively evaluate the performance of REFA relative to ULS-EFA under a variety of simulation conditions. By doing so, we aimed to better understand the conditions in which REFA is preferable to ULS-FA (or vice versa) for examining bifactor structures. We conclude this paper by providing substantive recommendations for researchers regarding the bifactor recovery capabilities of the two approaches.

## Background

### Exploratory Bifactor Models

The bifactor model originally introduced by Holzinger and Swineford (1937) was proposed to partition correlated factor structures into two components: the general factor, which takes into account the correlations among all factors; and group factors, which explicitly account for the unique influence of the specific domains left unaccounted for by the general factor. The factor structure of the classic bifactor model is as follows.

(1) $$\Lambda =\left(\begin{array}{ccc} \lambda_{11} & \lambda_{12} & 0\\ \lambda_{21} & \lambda_{22} & 0\\ \lambda_{31} & \lambda_{32} & 0\\ \lambda_{41} & 0 & \lambda_{43}\\ \lambda_{51} & 0 & \lambda_{53}\\ \lambda_{61} & 0 & \lambda_{63} \end{array}\right)$$

The first column of the matrix in equation (1) shows a general factor and the remaining columns represent group factors. As shown in this equation (1), the bifactor structure results from the constraint that each item loads on a general factor and only one group factor. The general factor accounts for the common variance among all items, while two group factors reflect additional common variance in the separate sets of domain-related items (Reise, 2012). For the *i*th item, [QSIImage] (general factor loading) and one of the *k* = 2, …, *m* values of [QSIImage] (group factor loadings) are freely estimated, while the remaining element is fixed to zero. The bifactor modeling has become a popular psychometric tool for multidimensional solutions due to its ability to incorporate both general and group factors in a single model (see Chen et al., 2006; Patrick et al., 2007; Pomplun, 2007).

Despite its advantages, the use of the bifactor model is often limited in some situations, due to the following reasons: First, when prior knowledge regarding the factor structure (derived from previous studies or field investigation) is absent, the bifactor model may yield misfit or biased parameter estimates because it is a specific form of confirmatory factor analysis. Second, even with sufficient information regarding the factor structure, unexpected dependencies among items may exist, which need to be captured by an additional dimensional structure (e.g., Furnham, 1990). Likewise, a restriction that fixes non-ignorable factor loadings for group factors to zero may yield biased results when applying the bifactor model.

All the aforementioned potential limitations highlight the importance of developing the bifactor model in an exploratory manner. Previous studies have proposed an exploratory form of the bi-factor model named exploratory bi-factor analysis (e.g., Schmid and Leiman, 1957; Jennrich and Bentler, 2011; Mansolf and Reise, 2016; Lorenzo-Seva and Ferrando, 2018). Among many approaches, the orthogonalization method proposed by Schmid and Leiman (1957) has become a commonly used exploratory bifactor analytic procedure. It allows researchers to estimate the exploratory form of the bifactor model in which items are free to load on a general factor and a set of group factors. The Schmid-Leiman technique transforms the second-order exploratory solution into an orthogonal solution containing a general factor and multiple group factors, which are uncorrelated with each other. Recently, researchers have relaxed this orthogonality restriction and developed bifactor solutions with oblique rotations, such as bi-quartimin and bi-geomin with the gradient projection algorithm (GPA) (see Jennrich and Bentler, 2011, 2012). Furthermore, Reise et al. (2011) suggested applying the orthogonal target rotation method for exploratory bifactor analysis by starting with a basic oblique rotation, then assigning items to factors according to that solution, creating a partially specified target matrix from said solution, adding a general factor to the target matrix (i.e., column of non-zero or unspecified elements), and rotating to said matrix. More extensive reviews of the history and development of exploratory bifactor analysis can be found in Lorenzo-Seva and Ferrando (2018) and Mansolf and Reise (2016). Exploratory bifactor analysis can be implemented in many software programs, including the R program (Bernaards and Jennrich, 2005; R Core Team, 2016), Mplus (Muthén and Muthén, 1998–2012), EQS (Bentler and Wu, 2002), and the SAS and SPSS macros (see Wolff and Preising, 2005).

### Regularized Exploratory Factor Analysis

When implementing the Schmid-Leiman procedure, researchers need to make decisions regarding two key issues - the factor extraction and the oblique factor rotation (see Reise et al., 2010). Because the results of this procedure are likely to be affected by the researchers’ choices of factor extraction and factor rotation methods, a deeper understanding of these decisions is needed. In Schmid-Leiman exploratory bifactor analysis, factor extraction decisions are especially critical for two reasons. First, the quality of group factor solutions in the Schmid-Leiman depends on how well a factor extraction method can provide unique variance estimates for the first-order factors. Second, there is no generally accepted oblique rotation method that researchers should follow (Browne, 2001); indeed, research has shown that all oblique rotation methods tend to provide comparable results (Fabrigar et al., 1999).

Though there are several factor extraction methods, ULS-FA was often preferred in studies involving small samples (e.g., de Winter et al., 2009). REFA was proposed in an attempt to mitigate the small sample size problem by incorporating the idea of regularization into a common factor model. It is considered as a valid alternative method for small samples because it only requires the iterative estimation of one parameter to find stable factor solutions. At this point, the body of research regarding the technical underpinnings of ULS in exploratory factor analysis is quite substantial (see Harman, 1976). Although REFA was proposed nearly a decade ago, this method is still novel to applied researchers in the field of behavioral science. In this section, we provide a gentle introduction to regularized exploratory factor analysis (see Jung and Takane, 2008 for more technical details), and then describe how to implement REFA for exploratory bifactor modeling in a stepwise fashion.

Regularization is considered as a useful technique for producing more accurate solutions in a wide range of multivariate data analysis. A popular application of regularization is ridge regression (Hoerl and Kennard, 1970), which is based on the prior knowledge that regression coefficients would never shift away from zero. We exploit this prior knowledge by imposing a regularization parameter on regression coefficients. This has the effect of shrinking regression estimates toward zero, thereby facilitating the production of more accurate solutions. This benefit of regularization should be more evident when sample sizes are small (e.g., Jung and Park, 2018).

The common factor model is equipped with both latent common and unique factors. Variance of each measured variable is made up of common variance due to common factors and unique variance due to unique factors. Part of unique variance represents random error due to unreliability or measurement error. One of the primary advantages of exploratory factor analysis is that it allows researchers to explicitly account for measurement error in an observed variable. From the viewpoint of a regularization framework, the common factor model may be expressed as

(2) $$X^{\prime }M^{\left(L\right)}\left(\lambda \right)X=X^{\prime }X+\lambda L$$

where *X* is an *N* by *P* matrix of observed variables and *M* is a generalized ridge metric matrix (Takane and Yanai, 2008). If *L* is set equal to Ψ, the diagonal matrix of unique variance, and the regularization parameter (λ) is fixed to -1, then we obtain *X*′*X* − Ψ. Here, *X*′*X* represents a square matrix that contains the variances and covariances (or correlations) associated with observed variables. The term *X*′*M*^(Ψ)^(−1) *X* can be formulated in terms of *S* − Ψ or *R* − Ψ, where *S*and *R*are sample covariance and correlation matrices, respectively. We perform an eigenvector decomposition of this matrix to find factor loading estimates.

In practice, however, unique variances are unknown *a priori* and must be estimated using appropriate statistical methods. Various iterative methods such as maximum likelihood estimation (e.g., Chen, 2003) have been routinely used to estimate unique variances in the common factor model. A number of approaches have been developed to produce unique variance estimates via non-iterative procedures, including the partitioned covariance estimator (PACE) option of comprehensive exploratory factor analysis programs and many others (e.g., Ihara and Kano, 1986; Jennrich, 1987). Using these techniques, *L* in equation (2) can be replaced with a tentative estimate of unique variances $\hat{L}$. True unique variances (Ψ) may be written as $\Psi =\lambda \hat{L}$. The smaller the value of λ, the greater the penalty that is placed on unique variance estimates, which will lead to their shrinkage. That is, true unique variances are assumed to be proportional to the tentative estimates of unique variances obtained by non-iterative procedures. In other words, REFA shrinks such initial estimates toward zero, thereby making it possible to produce more accurate results, as they are on average closer to the true population values.

A practical advantage of REFA is that given non-iterative estimation procedure, it contains only one free parameter of unique variances that needs to be estimated: it involves the estimation of λ (equal to proportion) only. For this reason, the method performs reasonably well with small samples. In addition, it has no risk of improper solutions. An optimal value of the regularization parameter is determined by minimizing various optimization criteria, such as the traditional maximum likelihood factor analysis. Given unique variance estimates, factor loadings can be obtained in closed form and non-iteratively (e.g., Jöreskog, 1977).

We now present a simple regularization technique for the Schmid-Leiman bifactor EFA. We developed a regularized extension of exploratory bifactor analysis that integrates the Schmid-Leiman orthogonalization with REFA. The newly proposed method, called regularized exploratory bifactor analysis, enables us to exhibit an exploratory bifactor structure with small sample sizes. The REFA bifactor method involves the following steps:

1. Reveal the underlying structure of the primary factors with REFA;
2. Perform an oblique factor rotation (e.g., quartimin);
3. Extract a second-order factor from the primary factor correlation matrix using REFA; and
4. Apply the Schmid-Leiman transformation to the second-order factor solution to obtain the loadings for each item on general and group factors that are orthogonal to each other.

The main benefit of exploratory bifactor analysis is to account for the unique contribution of group factors over and above the general factor. In the Schmid-Leiman procedure, an item’s loading on a group factor is simply its loading on the primary factor multiplied by the square root of the disturbance (the disturbance is the variance of the primary factor that remains even after the second-order factor is partialled out). Notably, the disturbance variance is calculated in the same manner as described above for the estimation of unique variances in REFA. The application of the proposed technique will yield more stable and interpretable estimates of loadings on group factors in small samples.

## Simulation Experiment

The primary goal of the simulation study was to compare the performance of REFA to that of ULS-FA in terms of bifactor structure recovery. A secondary goal was to evaluate the salience of a comprehensive set of important factors and their interactions in the factor recovery of these approaches. In the following section, we describe a variety of experimental conditions that we manipulated in the simulation, the data generation, the assessment criteria we evaluated, and the process of analysis.

### Design

The Monte Carlo simulation involved manipulating five experimental conditions: Sample Size (*N*), Variables per Factor ([QSIImage]), Number of Factors ([QSIImage]), Factor Loading (*l*), and Factor Correlation ([QSIImage]). These conditions are commonly adopted in simulation studies in the context of exploratory factor analysis (e.g., Garrido et al., 2013). Prior research has shown them to be meaningful conditions for differentiating the performance of factor analysis procedures (e.g., Velicer and Fava, 1998; MacCallum et al., 1999; Timmerman and Lorenzo-Seva, 2011).

The levels of the design factors were chosen such that they would represent the range of values encountered in empirical studies. The chosen ranges for the first three factors (*N, p/f*, and *f*) were essentially similar to those considered by Preacher and MacCallum (2002) in their simulations based on ULS-FA with small sample sizes. First, four levels of sample sizes—10, 20, 30, and 50 were simulated. These values were chosen to cover a broad range of empirical research settings from extremely small (10) to very small (50). A recent simulation study by de Winter et al. (2009) on applying ULS-FA demonstrated that even sample sizes smaller than 10 are sufficient for factor recovery. Budaev’s (2010) survey of the empirical literature reported that for factor analysis, the average number of observations was 64 (the minimum sample size = 4) in animal behavioral research. The number of variables per factor ([QSIImage]) was varied at two levels (4, 8) to systematically alter the degree of overdetermination. The overdetermination of a factor refers to the degree to which the factor is efficiently represented by a sufficient number of variables. Four variables per factor are considered as low overdetermination, and eight are considered as high overdetermination. Prior studies have found apparent associations between higher overdetermination and higher quality solutions in small samples (e.g., MacCallum et al., 1999). The number of group factors retained was either two or four, which would be typical of research practice. Next, the values of factor loading (*l*) were set to 0.40, 0.55, and 0.70, which, according to Comrey and Lee (1992), can be considered poor, good, and excellent, respectively. Finally, the values of factor correlation ([QSIImage]) were varied as 0.30, 0.50, and 0.70, corresponding to moderate, large, and very large correlation levels (de Winter et al., 2009).

### Data Generation

The full factorial design for the simulation resulted in a total of 144 factor combinations (4 *Sample Sizes* × 3 *Factor Loadings* × 3 *Factor Correlations* × 2 *Variables per Factors* × 2 *Number of Factors*). For each of the 144 different combinations, we generated multivariate normal data from[QSIImage], where [QSIImage] was the implied population correlation matrix derived from correlated-factors analysis formulation, using the Cholesky decomposition. For the generated datasets, ULS-FA may be susceptible to non-convergence or convergence to improper solutions such as a negative variance partially due to small samples, whereas no such problems occurred with REFA. For each combination of conditions described above, a total of 100 replications (unscreened samples) were first used to evaluate how serious the improper solutions problem is in ULS-FA. The likelihood of improper solutions with ULS-FA was found to be deleteriously affected by samples of 20 or fewer. For samples of 10, approximately 80% of the solutions by ULS-FA were improper. For samples of 20, the proportion of improper solutions dropped to 20%, whereas it did not exhibit any improper solutions with samples of 30 or larger. Extremely small samples can result in unweighted least squares estimation being unable to reach a solution. For the current study, however, any simulated sample that failed to converge within 200 iterations or yielded an improper solution was removed from further consideration to compare the two approaches in an impartial manner. The first 100 replications with proper solutions were maintained for each of the combinations of the design factors, finally resulting in 14400 samples for each approach. Previous studies generally regarded 100 replications as sufficient to produce consistent results in Monte Carlo simulation studies for exploratory factor analysis (e.g., MacCallum et al., 1999).

### Performance Measures

The practical utility of an exploratory bifactor analysis procedure depends heavily on its ability to recover the population factor structure. To assess the recovery of a bifactor structure under the approaches, two evaluation measures were calculated for each of the proper solutions: Tucker’s congruence coefficient ([QSIImage]) and the root mean squared error (RMSE). The measure of congruence represents a measure of factor similarity in a single study. On the other hand, one may think of RMSE as a measure of the accuracy of factor solutions over a long period within each experimental condition.

In the psychometric literature, the congruence coefficient has been commonly used to evaluate degrees of factor similarity (Lorenzo-Seva and ten Berge, 2006). Tucker’s congruence coefficient (Tucker, 1951) is a popular tool for evaluating the similarity of factor interpretations. The measure of congruence ([QSIImage]) is given by

(3) φ=trace(PF)′trace(PP′)trace(FF′),

where *P* and *F* represent a *p* by *m* population factor loading matrix and the corresponding sample matrix, respectively. Tucker’s index is typically computed after an orthogonal Procrustes rotation to prevent rotational indeterminacy. Higher congruence coefficient values indicate greater degrees of similarity between sample and population solutions.

Although high levels of factor similarity are required for effective interpretations, researchers should also consider whether item loading magnitudes are substantively meaningful. Accordingly, the usefulness of statistical procedures that produce relatively unstable parameter estimates is typically limited. To evaluate the accuracy of the estimation results, we computed the root mean squared error (RMSE) using the following equation:

(4) $$RMSE\left(g\right)={\left[\text{trace}\left(E^{\prime }E\right)/pm\right]}^{1/2},whereE=P-F.$$

Considering that the general factor and the group factors are uncorrelated, we computed RMSE values separately for each of the two types of factors.

### Analysis

In this study, we decomposed resulting sample correlation matrices using the following Schmid-Leiman bifactor procedure. First, a specified number of primary factors were extracted through either ULS-FA or REFA and rotated using oblique direct quartimin rotation (Bernaards and Jennrich, 2005). Next, a second-order factor was extracted from the primary factor correlation matrix using each of the two approaches. Finally, the second-order factor solution was transformed using the Schmid-Leiman orthogonalization to achieve a bifactor structure.

All computations for this study were carried out using MATLAB R2009a (The MathWorks Inc, 2009). We derived a ULS solution using an iterative principal factoring technique. The iterative process continued until the decrease in the criterion value was smaller than 10^–3^. This study recovered the order of the sample loadings in order to meet the highest overall Tucker’s congruence coefficient. The sign of the loadings can be recovered using a procedure suggested by Cliff (1966). In the end, for each proper sample solution, all performance measures were calculated using formulas (3) and (4).

## Simulation Results

In this section, we report the performance of ULS-FA and REFA in terms of recovering the population values for loadings. For each performance measure, we conducted a full-factorial six-way mixed ANOVA. A single within-subjects method factor was the estimation method (*M*, where *M* = ULS-FA or REFA). The between-subject data factors were the above-described five experimental conditions of the study. Table 1 presents the results regarding the capabilities of the two estimation methods. Given that our main focus was to compare the effectiveness of the two methods in terms of factor recovery, we discuss only the effects involving the method factor above the cut-off point. We omit other details of the ANOVAs due to space limitations.

**TABLE 1:** Results of Full Factorial 6-Way Repeated Measures ANOVAs.

Effects	*Df*	Congruence		RMSE (General factor)		RMSE (General factor)	
		*F*	η*^2^*	*F*	η*^2^*	*F*	η*^2^*
Within-subjects effects							
*M*	1	3538.57	0.20	14693.03	0.51	21285.50	0.60
M*L	2	161.73	0.02	799.49	**0.10**	737.34	**0.09**
M*P	1	344.04	0.02	1356.58	**0.09**	317.38	0.02
M*F	1	359.26	0.03	2719.85	**0.16**	563.29	0.04
M*R	2	23.85	0.00	27.78	0.00	3.30	0.00
M*N	3	141.74	0.03	11.62	0.00	147.67	0.03
M*L*P	2	10.81	0.00	7.27	0.00	4.31	0.00
M*L*F	2	38.53	0.01	32.64	0.01	13.18	0.00
M*L*R	4	2.91	0.00	2.20	0.00	1.90	0.00
M*L*N	6	56.04	0.02	10.57	0.00	4.63	0.00
M*P*F	1	170.42	0.01	555.65	0.04	915.51	**0.06**
M*P*R	2	1.50	0.00	21.87	0.00	8.01	0.00
M*P*N	3	35.19	0.01	25.94	0.01	184.47	0.04
M*F*R	2	1.12	0.00	82.23	0.01	5.46	0.00
M*F*N	3	16.47	0.00	0.94	0.00	81.32	0.02
M*R*N	6	5.47	0.00	2.09	0.00	3.17	0.00
M*L*P*F	2	7.05	0.00	21.87	0.00	42.05	0.01
M*L*P*R	4	2.31	0.00	1.75	0.00	0.70	0.00
M*L*P*N	6	13.94	0.01	25.43	0.01	22.04	0.01
M*L*F*R	4	2.82	0.00	7.70	0.00	1.35	0.00
M*L*F*N	6	16.30	0.01	11.97	0.01	19.54	0.01
M*L*R*N	12	1.40	0.00	1.39	0.00	1.06	0.00
M*P*F*R	2	10.55	0.00	10.86	0.00	3.61	0.00
M*P*F*N	3	9.12	0.00	51.43	0.01	702.54	**0.13**
M*P*R*N	6	2.32	0.00	2.31	0.00	2.46	0.00
M*F*R*N	6	1.30	0.00	2.20	0.00	1.54	0.00
M*L*P*F*R	4	1.66	0.00	1.33	0.00	0.40	0.00
M*L*P*F*N	6	5.07	0.00	41.01	0.02	29.74	0.01
M*L*P*R*N	12	2.00	0.00	1.66	0.00	2.00	0.00
M*L*F*R*N	12	0.66	0.00	0.56	0.00	1.45	0.00
M*P*F*R*N	6	6.69	0.00	0.98	0.00	7.60	0.00
M*L*P*F*R*N	12	0.90	0.00	0.49	0.00	1.41	0.00
Error (*M*)	14256						
Between-subjects effects							
Intercept	1	1563367.12	0.99	202283.98	0.93	595272.88	0.98
*L*	2	12535.99	0.64	3335.83	0.32	7847.60	0.52
*P*	1	3.59	0.00	3.00	0.00	249.69	0.02
*F*	1	3519.71	0.20	198.78	0.01	625.17	0.04
*R*	2	2.57	0.00	294.15	0.04	22.97	0.00
*N*	3	3892.86	0.45	6982.22	0.60	13639.73	0.74
L*P	2	28.70	0.00	5.56	0.00	0.37	0.00
L*F	2	32.12	0.00	71.42	0.01	186.11	0.03
L*R	4	7.41	0.00	13.11	0.00	35.81	0.01
L*N	6	50.55	0.02	54.55	0.02	8.70	0.00
P*F	1	66.34	0.01	21.02	0.00	88.05	0.01
P*R	2	1.15	0.00	2.62	0.00	1.12	0.00
P*N	3	29.31	0.01	5.04	0.00	24.20	0.01
F*R	2	2.45	0.00	66.92	0.01	6.88	0.00
F*N	3	8.82	0.00	0.88	0.00	24.50	0.01
R*N	6	4.60	0.00	20.53	0.01	34.14	0.01
L*P*F	2	11.60	0.00	5.66	0.00	5.74	0.00
L*P*R	4	1.39	0.00	1.79	0.00	1.27	0.00
L*P*N	6	6.59	0.00	0.66	0.00	6.74	0.00
L*F*R	4	3.52	0.00	1.76	0.00	6.45	0.00
L*F*N	6	28.54	0.01	3.50	0.00	5.70	0.00
L*R*N	12	2.04	0.00	2.60	0.00	3.15	0.00
P*F*R	2	0.00	0.00	0.73	0.00	0.11	0.00
P*F*N	3	6.91	0.00	0.74	0.00	7.18	0.00
P*R*N	6	2.03	0.00	1.00	0.00	1.97	0.00
F*R*N	6	0.60	0.00	3.19	0.00	1.25	0.00
L*P*F*R	4	1.43	0.00	0.67	0.00	1.58	0.00
L*P*F*N	6	0.26	0.00	0.09	0.00	4.12	0.00
L*P*R*N	12	1.84	0.00	1.91	0.00	1.94	0.00
L*F*R*N	12	3.18	0.00	1.24	0.00	4.88	0.00
P*F*R*N	6	0.63	0.00	1.20	0.00	2.27	0.00
L*P*F*R*N	12	0.86	0.00	0.95	0.00	1.20	0.00
Error	14256						

*M = method, L = factor loading, P = number of variables per factor, F = number of factors, R = factor correlation, and N = sample size. RMSE = root mean squared error. All F values are statistically significant (p < 0.05) except for those underlined. d.f. = degrees of freedom and = Effect Size. Interaction effects having η^2^ greater than 6% are shown in boldface.*

As Table 1 shows, most of the main and interaction effects were statistically significant due to the large number of observations, in addition to fitting all possible interactions in the ANOVA. For this reason, examining the effect size was crucial as well (e.g., Paxton et al., 2001). Following accepted practice for identifying a substantial effect, we focused on the main and interaction effects that have a partial eta-squared (η^2^) greater than 6%, which deserve further examination (MacCallum et al., 1999, for more details). According to Cohen’s (1988) guidelines regarding effect sizes, a value of 0.02 represents a small effect, 0.06 a medium effect, and 0.14 or greater a large effect.

### Measure of Congruence (QSIImage)

**FIGURE 1:**
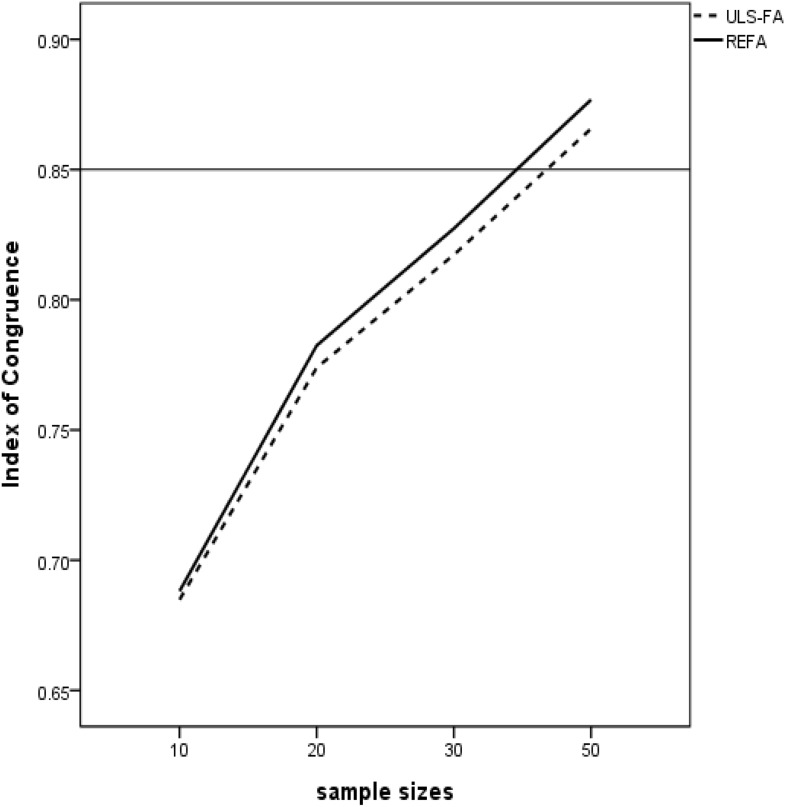
Two-way interaction between Method and Sample Size with congruence values as dependent variable (Horizontal bar = a criterion cutoff of 0.85).

As shown in Table 1, none of the two-way and higher order interactions with the analysis method in the coefficient of congruence were substantial enough for further examination; all of them had effect sizes below the cutoff of 6%. Following Cohen’s guidelines, nonetheless, nearly all the two-way interactions involving the sample size, the number of variables per factor, factor loadings, and the number of factors had non-negligible effect sizes, and these variables were also theoretically relevant determinants of factor recovery. We discuss the interaction of sample size by estimation method further due to its small effect size (η^2^ = 0.03), as the primary focus of our research is the ability of the two approaches in the small sample size condition. Figure 1 displays the average index of congruence obtained from the two approaches under the sample size condition. In general, REFA were associated with higher values of congruence coefficients (i.e., higher factor similarity) when compared to those obtained with ULS-FA. Lorenzo-Seva and ten Berge (2006) suggested the minimum 0.85 threshold for fair similarity. This means that when the sample size is 30 or greater, REFA might provide meaningful information about factor similarity.

### Root Mean Squared Error (RMSE): General Factor

**FIGURE 2:**
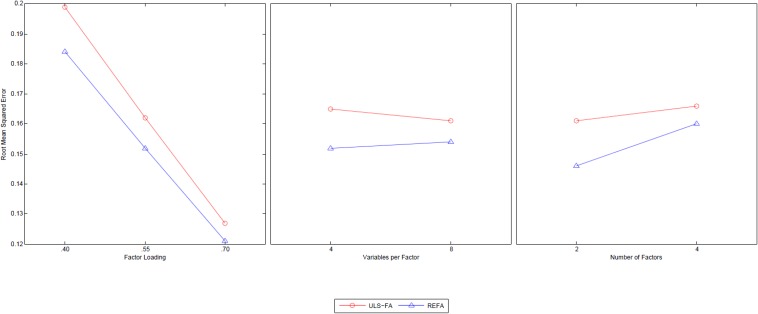
Two-way interactions of Method with Factor Loading, Variables per Factor, and Number of Factors with the RMSE of general factor as dependent variable.

The analysis method (η^2^ = 0.51) had a sufficiently large main effect. This suggests meaningful differences in the root mean squared error of general factor loadings between the two approaches (REFA = 0.15 and ULS-FA = 0.17). Moreover, three of the two-way interaction effects were statistically significant and achieved effect sizes larger than 6%: Method [QSIImage] Factor Loading (η^2^ = 0.10), Method [QSIImage] Variables per Factor (η^2^ = 0.09), and Method [QSIImage] Number of Factors (η^2^ = 0.16). Figure 2 shows each of these three interactions.

The findings displayed in Figure 2 clearly indicate that REFA is superior across the three meaningful conditions of factor loading, variables per factor, and number of factors. As the first column of Figure 2 shows, while we found a notable mean difference in the RMSE between the two methods with factor loadings of 0.4, this difference decreased as the factor loadings increased. Previous studies have reported that ULS-FA exhibits better performance for factor recovery in the presence of higher factor loadings (e.g., MacCallum et al., 1999). As the middle column shows, REFA produced a smaller RMSE (i.e., closer to true parameters) than ULS-FA across two levels of overdetermination. The average RMSE value for REFA under low overdetermination (four variables per factor) was slightly higher than that under high overdetermination (eight variables per factor). A decreasing tendency, however, emerged for ULS-FA: high overdetermination resulted in better factor recovery. Preacher and MacCallum’s (2002) simulation study identified the same pattern. As the last column shows, we found that REFA is clearly superior to ULS-FA with two group factors and equally accurate or slightly superior with four group factors. As the number of factors increased, the differences in the RMSE of the general factor loading estimates became negligible.

### Root Mean Squared Error (RMSE): Group Factors

The ANOVA for RMSE in the recovery of group factors revealed three noticeable interactions of interest: Method [QSIImage] Factor Loading (η^2^ = 0.09), Method [QSIImage] Variables per Factor [QSIImage] Number of Factors (η^2^ = 0.06), and Method [QSIImage] Variables per Factor [QSIImage] Number of Factors [QSIImage] Sample Size (η^2^ = 0.13). Although we found a sizable two-way interaction between method and factor loadings, we do not discuss this interaction below because it follows the same pattern as earlier results for the analysis of the interaction between these two design factors in the general factor. We describe the three-way interaction in detail later as it is nested within the four-way interaction.

We place particular emphasis on the four-way interaction for the following reasons: First, this higher-order interaction had the largest effect size worthy of further investigation. Second, it was produced by the interaction of the sample size with the three factors that had already appeared in the lower-order interactions. These results demonstrated the impact of sample size on the performance of the two approaches, which can be attributed to different procedures for the estimation of unique variance associated with each first-order factor.

**FIGURE 3:**
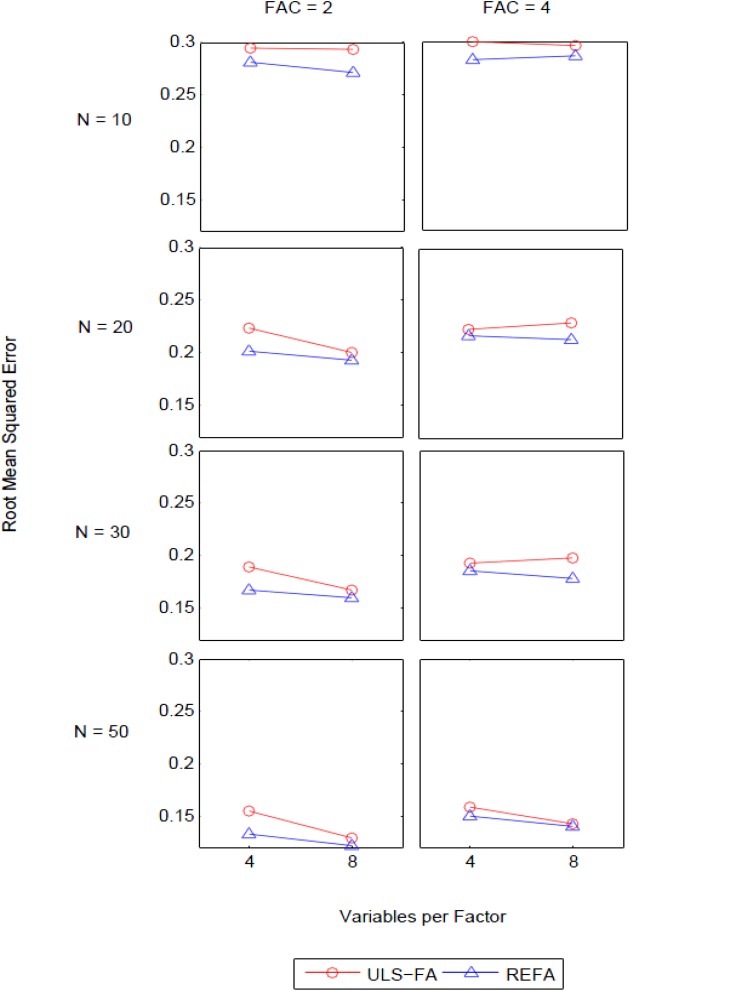
Four-way interactions of Method [QSIImage] Variables per Factor [QSIImage] Number of Factors [QSIImage] Sample Size with the RMSE of group factors as dependent variable (FAC = number of factors and N = sample size).

Figure 3 presents the four-way interaction of Method [QSIImage] Variables per Factor [QSIImage] Number of Factors [QSIImage] Sample Size. We found several intriguing characteristics with respect to group factor recovery. First, uniformly, REFA yielded lower RMSE than ULS-FA across all sample sizes. The two-way interaction of Method [QSIImage] Variables per Factor can be seen in the majority of the six blocks. In general, the difference in the RMSE between the two methods was more pronounced for weakly determined factors (here, four variables per factor) (see also Jung, 2013). Second, when few group factors were retained, REFA resulted in smaller RMSE under higher overdetermination (eight variables per factor) as compared to under lower overdetermination (four variables per factor) across all sample sizes. Of particular note is that ULS-FA performed similarly under the two levels of overdetermination when *N* = 10. However, this method experienced a steeper decline in the RMSE as sample sizes increased. This suggests that sample size plays a greater role in the performance of ULS-FA than in the performance of REFA. Overall, sample size and overdetermination are likely to have an important impact on good recovery of group factors. Third, as the number of group factors increased, however, there were relatively few differences in RMSE between the lower and higher levels of overdetermination for each approach across all sample sizes (except for *N* = 50). This finding suggests that, under this condition, sample size is likely to have little impact on the quality of results and good recovery can be obtained with relatively larger samples.

## Discussion

In this section, we discuss the implications of the simulation study and lay out guidelines for choosing between the two approaches. Researchers generally regard REFA as a viable alternative to ULS-FA in small-sample situations. However, to date, no study has examined the relative performance of these two methods in the context of exploratory bifactor analysis. Consequently, comprehensive Monte Carlo study was performed systematically to compare the relative performance of REFA and ULS-FA in several experimental conditions.

Our major findings are as follows: First, when factor loadings were poor, and thus communalities were low, REFA generally recovered loadings on general factor better than ULS-FA. When factor loadings were excellent, reflecting high communalities, ULS-FA recovered its loadings relatively well. The finding that ULS-FA performed well for a small sample coincides with the findings of previous studies, which have performed factor analyses with small sample sizes (e.g., MacCallum et al., 1999). Second, in general, REFA had smaller RMSEs of group factors than those of ULS-FA across all sample sizes. When few weakly determined factors were retained, REFA generally performed better in recovering loadings on group factors than ULS-FA. When a small number of factors were highly overdetermined, the differences in the RMSE between the two methods became negligible as sample sizes increased. However, in the case of extracting a large number of factors, the differences between them in the estimates of loadings on group factors were rather small regardless of levels of overdetermination. Finally, factor correlations did not lead to substantial differences in factor recovery between the two methods. We expected this result as the bifactor model relies on the assumption of relatively high correlation among factors in the estimation of parameters. These findings have important implications for researchers who may use exploratory bifactor analysis under the condition of small sample sizes.

### Implication for Practice

It is well known that exploratory factor analysis requires a relatively larger sample size to perform well, such as 100–200 observations. However, data sets with small samples are common in the various behavioral science disciplines such as comparative psychology and behavior genetics. Given the exigencies of applied research, behavioral scientists have called for the development of statistical methods that are effective in such traditionally difficult situations. This problem is likely to be exacerbated as the number of observed variables and latent variables increases. Recently, Jung and Takane (2008) have developed REFA via incorporating regularization methods into the common factor model. REFA has been widely used particularly in the comparative psychology literature (e.g., Tkaczynski et al., 2019). The current study expanded the literature by incorporating the idea of regularization into the bifactor model.

The present study provides a greater understanding of factor recovery capability of REFA and ULS-FA within the framework of exploratory bifactor modeling. First, the ability of REFA to perform relatively well with small samples, in particular in the case of a small number of underlying factors, is an important result since small sample sizes may be the rule rather than the exception, and researchers are more favorably inclined to develop efficient and parsimonious theories. Since ULS-FA is known to perform similarly to REFA with a large number of factors, there may be little reason to choose REFA over ULS-FA under the retention of large numbers of factors. Second, when researchers are less confident in selecting the correct number of factors, the choice of REFA or ULS-FA will likely be influenced by the degree of reliability of measurement. Guadagnoli and Velicer (1988) pointed out that factor scores are considered reliable if each factor has four or more loadings of at least 0.6 regardless of sample size. Researchers should take care to maintain reliable measures. If the measurement error is assumed to be small, the use of ULS-FA is justifiable. However, in the presence of a large amount of measurement error, REFA is recommended. Third, due to the greater likelihood of improper solutions with ULS-FA for extremely small samples of 10 and 20, researchers may consider using REFA over ULS-FA. Under such condition, however, factor similarity was poor with both of the two approaches. This can potentially misguide factor interpretation. Furthermore, they were associated with a large range of RMSE values. In sum, these findings suggest that the results obtained by REFA for samples of 10 and 20 should be discussed with caution in terms of accuracy and factor interpretation. When samples are 30 or larger, REFA tends to produce a bifactor solution reasonably well.

### Limitations

As with any study, the present study is not free of limitations. First, this study was designed to generate synthetic data within a continuous variable framework. The bifactor model is often estimated with ordinal-scale data using the ULS estimation procedure. Thus, it may be required to investigate the performance of REFA and ULS-FA with the sample matrix of ordinal-scale variables. Second, as is the case with all Monte Carlo simulation studies, the relative performance of each method in this study depended on the specific levels chosen for the experimental conditions. Although our simulation study took into account various experimental conditions that are frequently used in Monte Carlo simulation studies in the area of common factor analysis, the generated simulation conditions may not reflect the wide array of scenarios in empirical studies. For instance, population model misfit (e.g., due to minor factors) was not taken into account in the current study, even though it is inevitable to some extent in empirical studies and it may have harmful impact on factor recovery, particularly in the analysis of empirical data with a small number of observations. This signifies that the use of ULS-FA or REFA for small samples might lead to less optimal results with empirical data than suggested by the simulated data results. Thus, future studies should be conducted to fully investigate the relative performance of the two approaches under a wide range of conditions, in the presence of model misfit (e.g., MacCallum et al., 2001).

## Data Availability Statement

Requests to access the datasets should be directed to SJ, sunho.jung@khu.ac.kr.

## Author Contributions

SJ and DS contributed conception and design of the study. JP organized the database. SJ performed the statistical analysis and wrote the first draft of the manuscript. DS, JP, and SJ wrote sections of the manuscript. All authors contributed to manuscript revision, read and approved the submitted version.

## Conflict of Interest

The authors declare that the research was conducted in the absence of any commercial or financial relationships that could be construed as a potential conflict of interest.
